# Maternal Occupational Exposure to Polycyclic Aromatic Hydrocarbons: Effects on Gastroschisis among Offspring in the National Birth Defects Prevention Study

**DOI:** 10.1289/ehp.1104305

**Published:** 2012-02-13

**Authors:** Philip J. Lupo, Peter H. Langlois, Jennita Reefhuis, Christina C. Lawson, Elaine Symanski, Tania A. Desrosiers, Zeina G. Khodr, A.J. Agopian, Martha A. Waters, Kara N. Duwe, Richard H. Finnell, Laura E. Mitchell, Cynthia A. Moore, Paul A. Romitti, Gary M. Shaw, for the National Birth Defects Prevention Study

**Affiliations:** 1 Division of Epidemiology, Human Genetics and Environmental Sciences, University of Texas School of Public Health, Houston, Texas, USA; 2 Birth Defects Epidemiology and Surveillance Branch, Texas Department of State Health Services, Austin, Texas, USA; 3 National Center on Birth Defects and Developmental Disabilities, Centers for Disease Control and Prevention, Atlanta, Georgia, USA; 4 National Institute for Occupational Safety and Health, Centers for Disease Control and Prevention, Cincinnati, Ohio, USA; 5 Department of Epidemiology, University of North Carolina–Chapel Hill, Chapel Hill, North Carolina, USA; 6 Dell Pediatric Research Institute, Department of Nutritional Sciences, University of Texas, Austin, Texas, USA; 7 Department of Epidemiology, College of Public Health, University of Iowa, Iowa City, Iowa, USA; 8 Department of Pediatrics, Stanford School of Medicine, Palo Alto, California, USA

**Keywords:** birth defects, gastroschisis, maternal exposure, occupation, PAHs

## Abstract

Background: Exposure to polycyclic aromatic hydrocarbons (PAHs) occurs in many occupational settings. There is evidence in animal models that maternal exposure to PAHs during pregnancy is associated with gastroschisis in offspring; however, to our knowledge, no human studies examining this association have been conducted.

Objective: Our goal was to conduct a case–control study assessing the association between estimated maternal occupational exposure to PAHs and gastroschisis in offspring.

Methods: Data from gastroschisis cases and control infants were obtained from the population-based National Birth Defects Prevention Study for the period 1997–2002. Exposure to PAHs was assigned by industrial hygienist consensus, based on self-reported maternal occupational histories from 1 month before conception through the third month of pregnancy. Logistic regression was used to determine the association between estimated occupational PAH exposure and gastroschisis among children whose mothers were employed for at least 1 month during the month before conception through the third month of pregnancy.

Results: The prevalence of estimated occupational PAH exposure was 9.0% in case mothers (27 of 299) and 3.6% in control mothers (107 of 2,993). Logistic regression analyses indicated a significant association between occupational PAHs and gastroschisis among mothers ≥ 20 years of age [odds ratio (OR) = 2.53; 95% confidence interval (CI): 1.27, 5.04] after adjusting for maternal body mass index, education, gestational diabetes, and smoking. This association was not seen in mothers < 20 years (OR = 1.14; 95% CI: 0.55, 2.33), which is notable because although young maternal age is the strongest known risk factor for gastroschisis, most cases are born to mothers ≥ 20 years.

Conclusion: Our findings indicate an association between occupational exposure to PAHs among mothers who are ≥ 20 years and gastroschisis. These results contribute to a body of evidence that PAHs may be teratogenic.

Gastroschisis is a congenital malformation characterized by a herniation of viscera through an abdominal wall defect lateral to the umbilicus. The cause of gastroschisis is unknown; however, suggested mechanisms underlying this condition include vascular disruption (Werler et al. 2009) and failure of mesenchymal differentiation due to early teratogenic exposures (Feldkamp et al. 2007; Sadler and Feldkamp 2008). A recent increase in the prevalence of gastroschisis at birth has led to growing interest in identifying modifiable risk factors (Alvarez and Burd 2007; Benjamin et al. 2010; Mac Bird et al. 2009). Established risk factors for gastroschisis include young maternal age (< 20 years) and cigarette smoking (Mac Bird et al. 2009). Additionally, occupational exposures have been suggested as potential risk factors for gastroschisis (Herdt-Losavio et al. 2010; Mac Bird et al. 2009).

Assessing workplace exposures as risk factors for birth defects is of importance because > 95% of employed women in the United States remain employed during pregnancy (U.S. Census Bureau 2009). Furthermore, an increasing number of women are being exposed to potentially teratogenic chemicals in their jobs (Rice and Baker 2007). A prevalent group of toxic chemicals found in the workplace are polycyclic aromatic hydrocarbons (PAHs), which are formed during the incomplete burning of organic substances (Brandt and Watson 2003). Occupational settings where exposure to PAHs is likely to occur include oil and gas production, coal-fired and other power plants, and restaurants [Agency for Toxic Substances and Disease Registry (ATSDR) 1995; Sjaastad and Svendsen 2009].

Occupational exposure to PAHs has been associated with several adverse health outcomes, such as childhood cancer in the offspring of exposed mothers and bladder cancer (ATSDR 1995; Bosetti et al. 2007; Brandt and Watson 2003; Cordier et al. 1997; Hansen et al. 2008; Kogevinas et al. 2003). In mouse (Barbieri et al. 1986; Lambert and Nebert 1977; Shum et al. 1979), chick (Anwer and Mehrotra 1988), rat (Stark et al. 1989), and fish (Farwell et al. 2006; Incardona et al. 2004) models, PAHs have been shown to be reproductive toxicants, causing gastroschisis and a variety of other malformations. There is also growing evidence from human studies of adverse developmental effects from prenatal exposure to PAHs (Naufal et al. 2010; Ren et al. 2011). Despite this evidence, much work remains to be done in humans evaluating the association between maternal occupational PAH exposure and gastroschisis or other birth defects. Gastroschisis is of particular interest in relation to PAH exposure because of the established association between this defect and cigarette smoke (Feldkamp et al. 2008; Lammer et al. 2008; Mac Bird et al. 2009; Werler et al. 2009), which is a source of PAHs (Hearn et al. 2010). The objective of this study was to examine the association between maternal occupational exposure to PAHs and gastroschisis in offspring.

## Materials and Methods

*Study participants.* The study population included case and control infants from the National Birth Defects Prevention Study (NBDPS), with estimated dates of delivery between 1 October 1997 and 31 December 2002. Details of the NBDPS have been published elsewhere (Yoon et al. 2001). In brief, the NBDPS is a population-based case–control study of major structural birth defects. For the period 1997–2002, case infants with one or more congenital anomalies were ascertained through eight birth defect surveillance systems throughout the United States (Arkansas, California, Georgia, Iowa, Massachusetts, New Jersey, New York, and Texas) and included live births, stillbirths, and induced pregnancy terminations. Abstracted data for all case infants were reviewed by clinical geneticists using specific classification criteria, including standardized case definitions and confirmatory diagnostic procedures (Rasmussen et al. 2003). Infants and fetuses with single gene disorders or chromosomal abnormalities were excluded. Before inclusion in the NBDPS, gastroschisis cases whose clinical presentation suggested a limb–body wall complex or amniotic band sequence were excluded to reduce the potential for heterogeneity among the case group, because these cases are believed to have a different etiology (Werler et al. 2009). For this analysis, all gastroschisis cases were live births. Control infants (live-born infants without major structural birth defects) were randomly selected from birth certificates or birth hospitals, depending on study site. Case and control mothers completed a 1-hr computer-assisted telephone interview (CATI) between 6 weeks and 2 years after the estimated date of delivery; the interview included sections on maternal conditions and illnesses, lifestyle and behavioral factors, and multivitamin use. Before the interview began, the interviewer read a script to the mother and obtained verbal informed consent for her participation in the study. The Centers for Disease Control and Prevention Institutional Review Board (IRB), along with the IRBs for each participating state, approved the NBDPS. Additionally, for this analysis, approval was obtained from the IRB of the University of Texas Health Science Center at Houston.

We limited our analysis to case infants with a diagnosis of gastroschisis but included all control infants. A clinical geneticist (C.A.M.) reviewed the records of all infants with gastroschisis and classified each case as having either an isolated defect or multiple defects (if additional unrelated birth defects were present). Finally, case and control mothers were eligible for our analysis if they worked in part-time or full-time jobs (paid or volunteer) for at least 1 month from 3 months before conception through the end of pregnancy.

*Exposure assessment.* The NBDPS CATI includes occupation-related questions for jobs held for at least 1 month from 3 months before conception through the end of pregnancy. Information collected included job title, name of company or organization, service provided or product made by the company, main activities or duties, and machines used. Mothers reported the month and year for the start and stop date of each job, as well as the days per week and hours per day worked. Each job was coded for occupation and industry using the Standard Occupational Classification (SOC) System (U.S. Bureau of Labor Statistics 2001) and the North American Industry Classification System (U.S. Bureau of Labor Statistics 2009).

Expert industrial hygienists reviewed all jobs of mothers who reported any employment in order to estimate exposure to PAHs. This expert review strategy was based on an approach that had been previously developed and used in the Baltimore–Washington Infant Study (Jackson et al. 2004). Specifically, as part of the NBDPS occupational exposure assessment, industrial hygienists involved in the project participated in a training session before reviewing the job histories. During training, the industrial hygienists were given definitions of the exposure variables (e.g., exposure to any PAH in each job) and a sample set of 100 example jobs. Each industrial hygienist independently rated the 100 example jobs, and then all industrial hygienists worked together to examine the rationale and assumptions behind their rating decisions, including discussing mechanisms of exposure and modifying factors. This process was intended to help the industrial hygienists calibrate their ratings. After training was complete, two industrial hygienists, working independently and blinded to case–control status, reviewed occupational data reported during the CATI to determine a dichotomous (yes/no) rating of potential occupational exposure to PAHs for each job, as well as a confidence score for their rating [scale of 1 (not confident) to 4 (very confident)]. Discrepancies between the two industrial hygienists were resolved by a consensus conference that involved the original two industrial hygienists plus a third (Rocheleau et al. 2011). Specifically, during the consensus conference, industrial hygienists discussed each discrepant rating until all three agreed. If they could not come to agreement through discussion, they reviewed the literature to inform further discussion until agreement was reached.

For this analysis, we focused on potential exposures during the critical time window for the development of gastroschisis (i.e., the month before conception through the third month of pregnancy) (Selevan et al. 2000; Werler et al. 1992). Therefore, a woman was classified as exposed if she had one or more jobs during this critical window that were rated as exposed, and was classified as unexposed if all of her jobs during this critical window were rated as unexposed. Women who did not work during this period were not included in this analysis.

*Covariates.* Data for maternal characteristics that are generally accepted or suspected to be associated with gastroschisis risk (Mac Bird et al. 2009) were obtained from the CATI: maternal age at delivery (< 20 or ≥ 20 years); parity (0 or ≥ 1 previous births); maternal race/ethnicity (non-Hispanic white, non-Hispanic black, Hispanic, or other); maternal education (≤ 12 or > 12 years); gestational diabetes (yes/no); maternal use of supplements containing folic acid in the month before conception through the third month of pregnancy (yes/no); maternal alcohol use in the month before conception through the third month of pregnancy (yes/no); maternal smoking in the month before conception through the third month of pregnancy [nonsmoker, light (< 15 cigarettes/day), moderate (15–24 cigarettes/day), or heavy (≥ 25 cigarettes/day)]; secondhand smoke at home in the month before conception through the third month of pregnancy (yes/no); secondhand smoke at work in the month before conception through the third month of pregnancy (yes/no); and maternal prepregnancy body mass index (BMI). Maternal prepregnancy BMI (kilograms per square meter) was categorized according to the National Heart, Lung and Blood Institute cutoff points (Gilboa et al. 2010): underweight (< 18.5 kg/m^2^), average weight (18.5–24.9 kg/m^2^), overweight (25.0–29.9 kg/m^2^), and obese (≥ 30.0 kg/m^2^). Data on meat consumption (none or less than once, one to three times, four times, or more than four times per month) during the year before the pregnancy of interest was obtained from a Willett Food Frequency Questionnaire (58 food items) administered during the NBDPS CATI (Willett et al. 1987) to account for potential dietary sources of PAHs (Boers et al. 2005).

*Statistical analysis.* Frequency distributions of maternal demographic and behavioral factors were tabulated for case and control infants. The crude odds ratio (OR) and 95% confidence interval (CI) was estimated for the association between each maternal factor and gastroschisis. Frequency distributions of the SOC major job groups (*n* = 23) were tabulated for mothers of cases and controls, stratified by occupational PAH exposure status. Each job was mapped to one of these 23 SOC job groups. Additionally, we assessed differences in mean time to interview using Student’s *t*-test for case and control mothers, as well as exposed and unexposed mothers, because the time lapse between estimated date of delivery and interview ranged from 6 weeks to 2 years. Unconditional logistic regression was used to calculate crude and adjusted ORs and 95% CIs to estimate the association between maternal occupational exposure to PAHs and the odds of gastroschisis in offspring. Results were stratified on maternal age (< 20 vs. ≥ 20 years), because young maternal age is one of the strongest risk factors for gastroschisis (Mac Bird et al. 2009), and associations with other risk factors (e.g., maternal smoking) appear to vary by maternal age (Werler et al. 2009). Additionally, we stratified our results on maternal smoking in an attempt to assess if exposure to cigarette smoke, a source of PAHs and other toxicants, modified the relationship between occupational PAH exposure and gastroschisis (Hearn et al. 2010). We also conducted two sensitivity analyses: First, we excluded women who did not have a job with a confidence rating of 4 (i.e., jobs with the highest confidence score) in an attempt to minimize exposure misclassification (17 case mothers and 56 control mothers); second, we excluded 24 cases with multiple defects to reduce potential etiologic heterogeneity. Variables were incorporated as confounders in the final models if inclusion resulted in a ≥ 10% change in the estimate of effect between maternal occupational exposure to PAHs and the odds of gastroschisis in offspring in the baseline model. All analyses were conducted using Intercooled Stata (version 10.1; StataCorp LP, College Station, TX).

## Results

Participation in the NBDPS was 71% among gastroschisis case mothers and 68% among control mothers. Of the 418 case mothers and 4,116 control mothers (*n* = 4,534) included in the NBDPS for the period 1997–2002, 73% were employed for at least 1 month during the critical window of exposure (the remaining 37% reported no job during the critical window), leaving 299 gastroschisis case infants and 2,993 control infants (*n* = 3,292) eligible for this analysis. Selected maternal characteristics are summarized by case–control status, along with the crude OR and 95% CI for each factor and gastroschisis, in Table 1. Compared with control mothers, mothers of cases were less likely to be ≥ 20 years of age, to be obese versus having normal BMI, or to have one or more previous births, an education level beyond high school, or gestational diabetes; and they were more likely to be light, moderate, or heavy smokers, to be exposed to secondhand smoke at home or work, and to have three or more jobs in the month before conception through the third month of pregnancy. There were no significant differences in the mean time to interview between case and control mothers (11.3 vs. 10.3 months, *p* = 0.32) or between PAH-exposed and unexposed mothers (11.3 vs. 11.4 months, *p* = 0.67).

**Table 1:** Distribution of maternal factors among employed^a^ mothers of gastroschisis case infants and control infants, NBDPS, 1997–2002 [n (%)].

Characteristic	Cases (n = 299)	Controls (n = 2,993)	OR (95% CI)
Age (years)						
< 20		106 (35.45)		240 (8.02)		1.00 (referent)
≥ 20		193 (64.55)		2,753 (91.98)		0.16 (0.12, 0.21)
Prepregnancy BMI (kg/m2)						
Underweight (< 18.5)		26 (8.81)		153 (5.22)		1.35 (0.87, 2.10)
Normal weight (18.5–24.9)		211 (71.53)		1,676 (57.16)		1.00 (referent)
Overweight (25–29.9)		52 (17.63)		664 (22.65)		0.62 (0.45, 0.85)
Obese (≥ 30)		6 (2.03)		439 (14.97)		0.11 (0.05, 0.25)
Parity						
0		196 (65.55)		1,331 (44.49)		1.00 (referent)
≥ 1		103 (34.45)		1,661 (55.51)		0.42 (0.33, 0.54)
Race/ethnicity						
Non-Hispanic white		177 (59.20)		1,940 (64.97)		1.00 (referent)
Non-Hispanic black		23 (7.69)		377 (12.63)		0.67 (0.43, 1.05)
Hispanic		77 (25.75)		528 (17.68)		1.60 (1.20, 2.12)
Other		22 (7.36)		141 (4.72)		1.71 (1.06, 2.75)
Education (years)						
≤ 12		189 (63.64)		1,037 (34.71)		1.00 (referent)
> 12		108 (36.36)		1,951 (65.29)		0.30 (0.24, 0.39)
Gestational diabetes						
No		290 (98.64)		2,804 (95.96)		1.00 (referent)
Yes		4 (1.36)		118 (4.04)		0.33 (0.12, 0.89)
Folic acid supplement useb						
No		181 (60.54)		1,402 (46.84)		1.00 (referent)
Yes		118 (39.46)		1,591 (53.16)		0.57 (0.45, 0.73)
Alcohol useb						
No		161 (54.03)		1,661 (55.72)		1.00 (referent)
Yes		137 (45.97)		1,320 (44.28)		1.07 (0.84, 1.36)
Smokingb						
Nonsmoker		186 (62.21)		2,378 (79.45)		1.00 (referent)
Light (< 15 cigarettes/day)		75 (25.08)		423 (14.13)		2.27 (1.70, 3.02)
Moderate (15–24 cigarettes/day)		30 (10.03)		159 (5.31)		2.41 (1.59, 3.66)
Heavy (≥ 25 cigarettes/day)		8 (2.68)		33 (1.10)		3.10 (1.41, 6.81)
Secondhand smoke at homeb						
No		199 (66.56)		2,456 (82.11)		1.00 (referent)
Yes		100 (33.44)		535 (17.89)		2.31 (1.78, 2.99)
Secondhand smoke at workb						
No		203 (67.89)		2,412 (80.83)		1.00 (referent)
Yes		96 (32.11)		572 (19.17)		1.99 (1.54, 2.58)
Meat consumptionc						
None or less than once a month		43 (14.38)		408 (13.66)		1.00 (referent)
One to three times a month		88 (29.43)		758 (25.38)		1.10 (0.75, 1.62)
Four times a month		77 (25.75)		893 (29.90)		0.82 (0.55, 1.21)
More than four times a month		91 (30.43)		928 (31.07)		0.93 (0.64, 1.36)
No. of jobs helda						
1		244 (81.61)		2,661 (88.91)		1.00 (referent)
2		45 (15.05)		312 (10.42)		1.57 (1.12, 2.21)
≥ 3		10 (3.34)		20 (0.67)		5.45 (2.52, 11.78)
aEmployed for at least 1 month from the month before conception through the third month of pregnancy. bOne month before conception through the third month of pregnancy. cIn the year before pregnancy.						

The exposure assessment yielded few discordant ratings between industrial hygienists in exposure assignment. Specifically, 250 mother jobs were discordant for the estimated PAH exposure rating among 12,492 (2%) included in the entire NBDPS (i.e., all cases and controls included in the NBDPS occupational PAH exposure assessment) (Rocheleau et al. 2011).

Table 2 displays the distributions of jobs within the 23 SOC major job groups held by mothers of cases and controls stratified by exposure status. Jobs in the sales and related occupations SOC job group were the most frequent held jobs among exposed case mothers (13 of 28 individual jobs held by exposed case mothers during the critical window), including 12 jobs as cashiers in fast food restaurants. The second largest SOC job group with PAH exposure among exposed case mothers was food preparation and serving related occupations (*n* = 11). The most common jobs among exposed control mothers were in food preparation and serving related occupations (52 of 109 individual jobs), followed by sales and related occupations (*n* = 32).

**Table 2:** Distributions of SOC major job groups held by mothers of gastroschisis case infants and control infants by estimated occupational PAH exposure status, NBDPS, 1997–2002.

Casesa			Controlsa		
SOC major group	Exposed	Unexposed	Exposed	Unexposed
Managementb		1		9		3		319
Business and financial operations		0		10		0		148
Computer and mathematical		0		2		0		52
Architecture and engineering		0		2		1		11
Life, physical, and social science		0		2		0		43
Community and social services		0		1		0		68
Legal		0		1		0		33
Education, training, and library		0		5		0		291
Arts, design, entertainment, sports, and media		0		5		0		50
Health care practitioners and technical		0		11		0		275
Health care support		0		17		1		167
Protective service`		0		1		0		25
Food preparation and serving related		11		53		52		230
Building and grounds cleaning and maintenance		0		8		1		73
Personal care and service		1		40		7		158
Sales and relatedc		13		71		32		444
Office and administrative support		0		92		0		746
Farming, fishing, and forestry		1		8		2		49
Construction and extraction		0		0		0		5
Installation, maintenance, and repair		0		1		1		5
Production		1		21		6		151
Transportation and material moving		0		9		3		77
Military specific		0		0		0		4
aIndividuals may be represented more than once if multiple jobs were held during the critical window of exposure (i.e., 1 month before conception through the third month of pregnancy). bExposed participants include managers in restaurants. cExposed participants include cashiers in fast food restaurants.								

Overall, the prevalence of estimated occupational PAH exposure was 9% in case mothers (27 of 299) and 4% in control mothers (107 of 2,993), and there was a significant (*p* < 0.001) crude association between estimated maternal occupational exposure to PAHs and gastroschisis in offspring (OR = 2.68; 95% CI: 1.72, 4.16) (Table 3). When we restricted our analysis to those mothers who had jobs rated with high confidence (*n* = 282 case mothers; *n* = 2,937 control mothers), the crude association was similar to that obtained in the full group (OR = 2.63; 95% CI: 1.68, 4.11). Additionally, there was a significant association (*p* = 0.03) between estimated maternal occupational exposure to PAHs and gastroschisis in offspring after adjusting for maternal age, BMI, education, gestational diabetes, maternal smoking, and study center (OR = 1.75; 95% CI: 1.05, 2.92).

**Table 3:** Crude and adjusted associations between estimated maternal occupational exposure to PAHs and gastroschisis in offspring, overall and stratified by maternal smoking and age, NBDPS, 1997–2002.

Exposure status	Cases [n (%)]	Controls [n (%)]	Crude OR (95% CI)	Adjusted ORa (95% CI)
No PAH exposure		272 (90.97)		2,886 (96.42)		1.00 (referent)		1.00 (referent)
PAH exposure		27 (9.03)		108 (3.58)		2.68 (1.72, 4.16)		1.75 (1.05, 2.92)
Maternal smoking								
No								
No PAH exposure		169 (90.86)		2,305 (96.97)		1.00 (referent)		1.00 (referent)
PAH exposure		17 (9.14)		72 (3.03)		3.22 (1.86, 5.59)		1.82 (0.94, 3.51)
Yes								
No PAH exposure		103 (91.15)		581 (94.32)		1.00 (referent)		1.00 (referent)
PAH exposure		10 (8.85)		35 (5.68)		1.61 (0.77, 3.36)		1.16 (0.51, 2.66)
Age (years)								
< 20								
No PAH exposure		92 (86.79)		210 (87.50)		1.00 (referent)		1.00 (referent)
PAH exposure		14 (13.21)		30 (12.50)		1.07 (0.54, 2.10)		1.14 (0.55, 2.33)
≥ 20								
No PAH exposure		180 (93.26)		2,676 (97.20)		1.00 (referent)		1.00 (referent)
PAH exposure		13 (6.74)		77 (2.80)		2.51 (1.37, 4.60)		2.53 (1.27, 5.04)
aAdjusted for maternal age, BMI, education, gestational diabetes, maternal smoking, and study center.								

Although the association between maternal occupational exposure to PAHs and gastroschisis in offspring among mothers who were < 20 years of age was not significant (adjusted OR = 1.14; 95% CI: 0.55, 2.33), there was a significant association among mothers ≥ 20 years of age (OR = 2.53; 95% CI: 1.27, 5.04) after adjusting for maternal BMI, education, gestational diabetes, maternal smoking, and study center (variables associated with gastroschisis and maternal occupational exposure) (Table 3). The association among mothers who were ≥ 20 years of age was similar when we restricted the analysis to mothers with jobs that were rated with high confidence (adjusted OR = 2.67; 95% CI: 1.34, 5.34). When results were stratified on maternal smoking, the association was stronger among nonsmoking mothers than among those who smoked at any time during the month before conception through the third month of pregnancy (Table 3). However, this difference was minimized after adjusting for maternal age, BMI, education, gestational diabetes, and study center (nonsmokers: OR = 1.82; 95% CI: 0.94, 3.51; smokers: OR= 1.16; 95% CI: 0.51, 2.66). Finally, all analyses were repeated among isolated case infants (*n* = 275) and control infants, and there was no difference in our results (crude OR = 2.70; 95% CI: 1.72, 4.23).

## Discussion

We observed an association between estimated maternal occupational exposure to PAHs and gastroschisis in offspring. However, although case and control mothers < 20 years of age were more likely to be classified as exposed (13.2% and 12.5%, respectively) than were older case and control mothers (6.7% and 2.8%, respectively), the association was limited to women ≥ 20 years of age. Other factors have also been reported to be associated with gastroschisis in the children of older women but not younger women. For instance, results from the NBDPS and a case–control study in Utah both suggest that the association between direct maternal smoking is stronger in women ≥ 20 years of age than in younger women (Feldkamp et al. 2008; Werler et al. 2009). Assuming older smokers have smoked for more years, it has been suggested that longer duration of smoking might contribute to uterine vascular damage (Suzuki et al. 1980), which in turn can lead to the development of gastroschisis (Werler et al. 2009). This could also be the case for prolonged exposure to occupational PAHs, although long-term information on maternal occupation before conception was not available for this analysis. Alternatively, the underlying mechanisms that produce gastroschisis among young mothers may differ from those that produce gastroschisis in the children of older mothers. Lastly, the difference in the magnitude of the effect measure estimates between younger and older mothers could be attributable to between-job exposure variability because exposed case mothers ≥ 20 years of age were more likely to be cooks, whereas exposed case mothers < 20 years of age were more likely to be restaurant cashiers. Cooks are likely to have a greater intensity of exposure to PAHs because of close proximity to high-temperature cooking compared with cashiers in the same restaurant.

Because PAHs are lipophilic, they readily penetrate cellular membranes (including the placenta) (ATSDR 1995). During PAH metabolism, enzymatic activity can result in the formation of reactive intermediates that covalently bind to DNA, forming adducts. DNA adducts have been shown to result in a spectrum of cellular mutations that may be teratogenic (Wells et al. 2010). PAH–DNA adducts have been isolated not only in adult tissues but also in placental tissues, amniotic fluid, and umbilical cord blood (Arnould et al. 1997; Madhavan and Naidu 1995; Ravindra et al. 2001). Furthermore, there is some evidence that occupational PAH exposure is associated with PAH–DNA adduct formation. For instance, a study by Perera et al. (1994) demonstrated that foundry workers with low-level exposure to PAHs had detectable levels of PAH–DNA adducts; however, a review by Brandt and Watson (2003) indicated that associations between measured PAH exposure and PAH–DNA adducts is equivocal. PAHs have been shown to be developmental toxicants in animal models, causing a range of birth defects (Anwer and Mehrotra 1988; Barbieri et al. 1986; Farwell et al. 2006; Incardona et al. 2004; Shum et al. 1979; Wassenberg and Di Giulio 2004; Wassenberg et al. 2005). To our knowledge, there have been only two other human studies of PAHs and birth defects (both case–control studies in China assessing neural tube defects). In a study by Naufal et al. (2010), PAH concentrations measured in venous blood samples were significantly (*p* < 0.05) higher in case mothers compared with control mothers. In a study by Ren et al. (2011), which included part of the same population as the Naufal et al. (2010) study, placental concentrations of PAHs were significantly higher (*p* < 0.001) in case placentas than in controls.

Our findings must be considered in light of certain limitations. The main limitation is related to the occupational exposure assessment. Although our approach relied on expert industrial hygienist consensus, there is still a potential for misclassification when assigning exposure based on questionnaire responses about jobs held. In an attempt to limit bias due to exposure misclassification, we restricted our analysis to those mothers with jobs that were rated with the highest confidence in the exposure assessment and found our results were similar. Furthermore, our approach is superior to a strategy that relies solely on maternal self-report of PAH exposure, where knowledge of PAH exposure is likely to be limited (Olsson et al. 2010). Although the use of personal monitoring or biomarkers of exposure would be preferred, these data are typically unavailable in population-based studies of birth defects, because these outcomes, although clinically significant, are relatively rare (e.g., the prevalence of gastroschisis is ~ 5 per 10,000 births) (Benjamin et al. 2010) and often not assessed in the context of prospective cohort studies (Yoon et al. 2001). Another limitation related to the occupational exposure assessment is the lack of information on intensity and frequency of exposure, which limits inferences about between- and within-job exposure variability and precludes exposure–response analyses.

A limitation with this and other case–control studies is the potential for recall bias. Because occupational PAH exposure was based on expert assessment rather than self-report, this may be less of a problem for our study (Jackson et al. 2004; Rocheleau et al. 2011). Furthermore, the impact of recall bias appears to be minimal in the NBDPS for many important risk factors, such as maternal smoking (MacLehose et al. 2009). The absence of information on environmental sources of PAHs is also a potential limitation, but occupational exposures are generally higher than those found in the environment (Brandt and Watson 2003). Additionally, we evaluated potential confounding by direct and secondhand smoke and meat consumption, which are important sources of environmental PAHs (Boers et al. 2005; Hansen et al. 2008). Finally, although we controlled for many measured maternal factors, there is still potential confounding by unidentified factors (i.e., unmeasured factors that have not been established as risk factors for gastroschisis). For instance, because most exposed women worked in food preparation or restaurant-related occupations, there may be some factor related to these occupations that is associated with both gastroschisis and PAH exposure. However, we attempted to adjust for several factors that may be associated with employment in these occupations (e.g., maternal age, education).

Strengths of this study include the use of data from the NBDPS, the largest population-based case–control study exploring risk factors for birth defects, which has an extensive occupational PAH exposure assessment available for study participants from 1997 through 2002. As part of the NBDPS, we also had information on potentially important confounding factors such as maternal nutrition, prepregnancy BMI, and smoking. Additionally, the case classification undertaken by NBDPS clinical geneticists to exclude cases due to single gene disorders or chromosomal abnormalities or those that are part of a limb–body wall complex or amniotic band sequence, resulted in a more homogeneous gastroschisis case group. Specifically, the exclusion of cases with known causes (e.g., single gene disorders) reduces the potential for etiologic heterogeneity in studies of birth defects (Khoury et al. 1982a, 1982b).

## Conclusions

To our knowledge, this study provides the first reported assessment of the potential association between estimated maternal occupational exposure to PAHs and gastroschisis in offspring. Our analyses indicated that maternal occupational PAH exposure during early pregnancy was associated with an increased odds of gastroschisis in the offspring of women ≥ 20 years of age, but not the offspring of younger women (i.e., < 20 years of age), which is notable because although young maternal age is the strongest known risk factor for gastroschisis, most cases are born to mothers ≥ 20 years. Future investigations of PAHs and gastroschisis could be improved by incorporating additional measures of exposure (e.g., biomarker data) and information on maternal and fetal genotypes related to PAH metabolism (Sanyal and Li 2007; Shimada 2006; Wassenberg and Di Giulio 2004; Wassenberg et al. 2005; Whyatt et al. 1998).
